# Supplementary material for: Integration of machine learning XGBoost and SHAP models for NBA game outcome prediction and quantitative analysis methodology
Source: PLoS One. 2024 Jul 23;19(7):e0307478. doi: 10.1371/journal.pone.0307478 (PMC11265715; doi:10.1371/journal.pone.0307478)
Supplement: Attachment — Submitted filename: Review of PONE-D-24-12722.docx [file pone.0307478.s001.docx]

Review of “Integration of machine learning XGBoost and SHAP models for NBA game outcome prediction and quantitative analysis methodology”

The manuscript is well-written, and the topic is also interesting. The data-driven analysis using ML tools is also rigorous. I have a few comments that the author should consider in a revised manuscript:

1. The authors primarily focus on the Basketball dataset. However, I feel the methods should be generalizable to other sports like Hockey and Football.
2. Figure 6 is not at all clear. So what are the feature importance of the feature variables.
3. In your work, you have basically compared the various ML algorithms and based on a certain metric, the xGBoost is chosen. This is what you do in a Hackathon. But you need to tell a story with the Data. I would appreciate it if you first conducted an exploratory analysis of the data with some interesting plots. You have given descriptive statistics, but that’s not enough. You should also present the significance of the pairwise correlations.
4. Finally, run some robustness tests using regression approaches so that we can get the significance of the independent variables and the interpretation of the coefficients will also be easier.
